# Supplementary material for: Long non-coding RNA-dependent mechanism to regulate heme biosynthesis and erythrocyte development
Source: Nat Commun. 2018 Oct 22;9:4386. doi: 10.1038/s41467-018-06883-x (PMC6197277; doi:10.1038/s41467-018-06883-x)
Supplement: Supplementary file 1 — Supplementary Information [file 41467_2018_6883_MOESM1_ESM.pdf]

Supplementary Information

Long Non-Coding RNA-Dependent Mechanism to Regulate Heme Biosynthesis and Erythrocyte Development

Liu et al.

Supplementary Figure 1

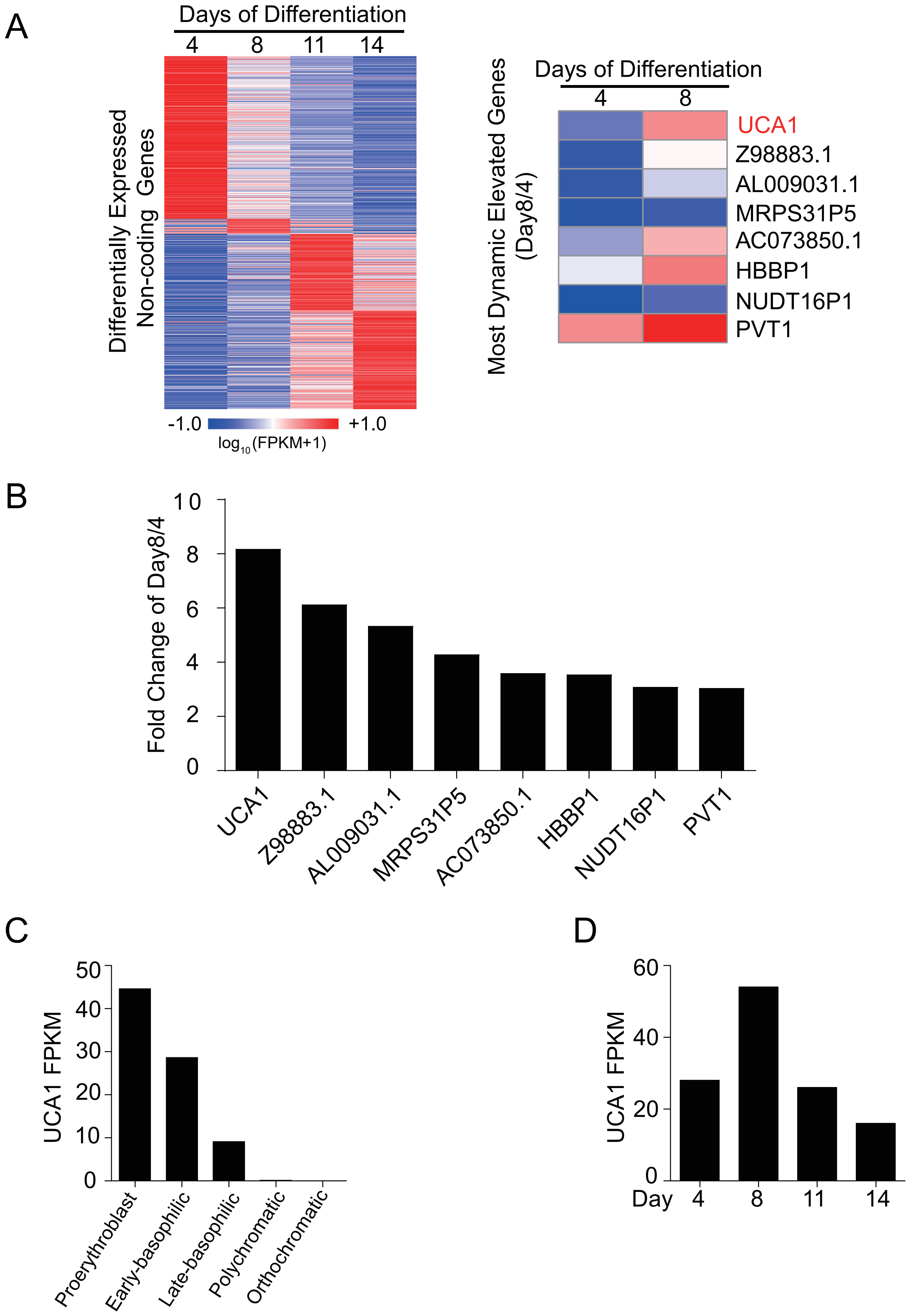

Supplementary Figure 1. Differential UCA1 expression during human erythroid differentiation (A) The heatmap on the left depicts ncRNA expression profiles with the mean expression value from two replicates. The ncRNAs were defined as genes excluding all protein coding ones from human GENCODE (version 27) annotation. The differential ncRNAs were identified by a pairwise comparison with Padj < 0.05 between any two differentiation stages [(D4 vs 8) U (D4 vs 11) U (D4 vs 14) U (D8 vs 11) U (D8 vs 14) U (D11 vs 14)] with an FPKM > 0.1 (average of two replicates) at any differentiation stage (D4 U D8 U D11 U D14). The right heatmap displays the top 8 ncRNAs which are most abundantly or dynamically expressed from day 4 to day 8 differentiation [Padj < 0.05 and an FPKM > 1 (average of two replicates) at any differentiation stage]. (B) The expression fold change of the top 8 ncRNAs between days 4 and 8 of the primary human erythroid cell differentiation culture. (C, D) data mined of UCA1 expression from FACS-sorted, stage-specific erythroid cells differentiated from cord blood $CD34^+$ cells (C) or from adult human peripheral blood $CD34^+$ cells (D).

Supplementary Figure 2

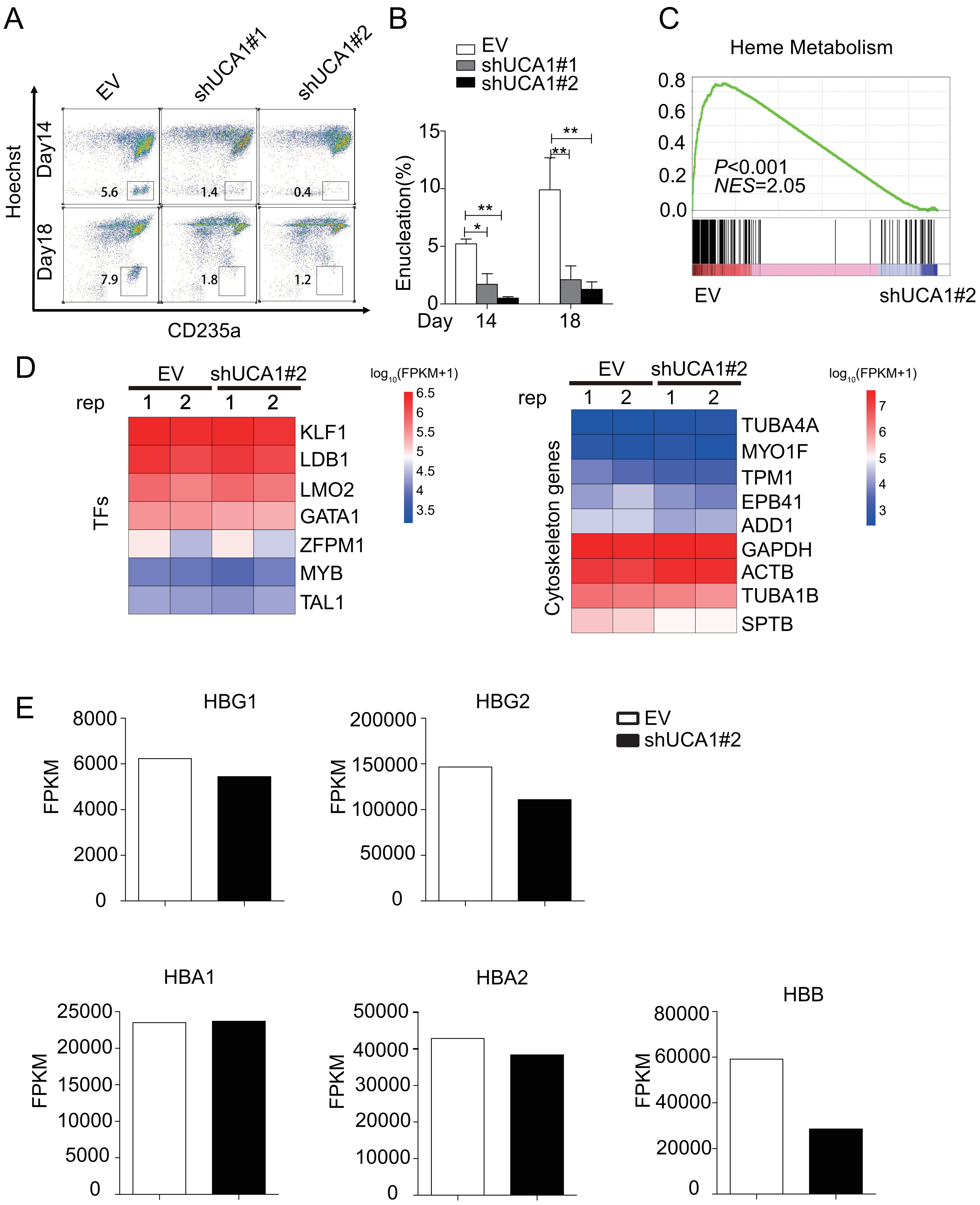

Supplementary Figure 2. Erythroid maturation is impaired after UCA1 depletion (A, B) Erythrocyte enucleation was quantified by FACS with Hoechst 33342 and CD235a staining (A). The graph depicts the percentage of enucleated cells (B). $n = 3$ independent experiments. Error bars represent SEM. P values were determined by Student's t test. *$P < 0.05$, **$P < 0.01$. (C) GSEA reveals enrichment of heme metabolism related genes after UCA1 knockdown. Normalized enrichment scores (NES) and P values are indicated. (D) The heatmap depicts the expression of key erythroid transcription factors and cytoskeletal genes after UCA1 depletion. (E) Globin expression (FPKM) was shown after *UCA1* depletion by RNA-seq.

Supplementary Figure 3

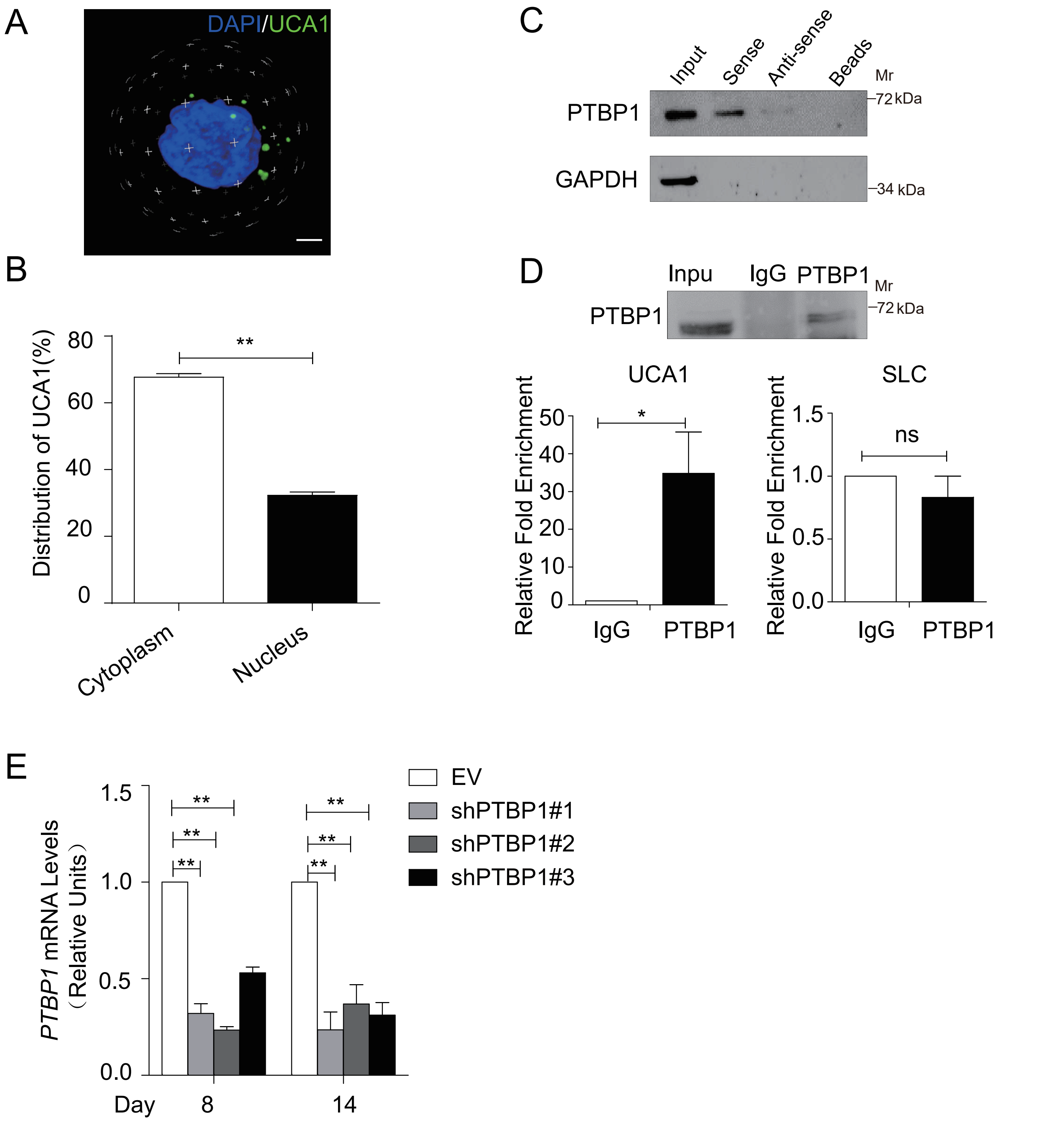

Supplementary Figure 3. PTBP1 protein interacts with UCA1 RNA in AraC-induced K562 cells (A, B) Representative 3D image of UCA1 RNA localization by RNA-FISH in primary erythroid cells differentiated *ex vivo* for 8 days (A). Scale bar = 5 μm. Quantification of UCA1 RNA distribution in cytoplasm and nucleus of erythroid cells (n = 100 cells from 3 independent experiments) (B). (C) RNA pull-down assay using *in vitro* transcribed UCA1 showed the physical interaction of UCA1 and PTBP1 in AraC-induced K562 cells. (D) PTBP1 RIP assay shows interactions between PTBP1 and UCA1 in AraC-induced K562 cells. WB showed PTBP1 immunoprecipitation (top). The relative fold enrichment of UCA1 compared to IgG was determined by qRT-PCR (bottom). SLC was used as a negative control. (E) Expression of *PTBP1* mRNA was quantitated by qRT-PCR after shRNA mediated PTBP1 knockdown in day 8 differentiated primary erythroid cells. *18S* rRNA was used for internal control. n = 3 independent experiments. Error bars represent SEM. P values were determined by Student's t test. *$P < 0.05$, **$P < 0.01$.

Supplementary Figure 4

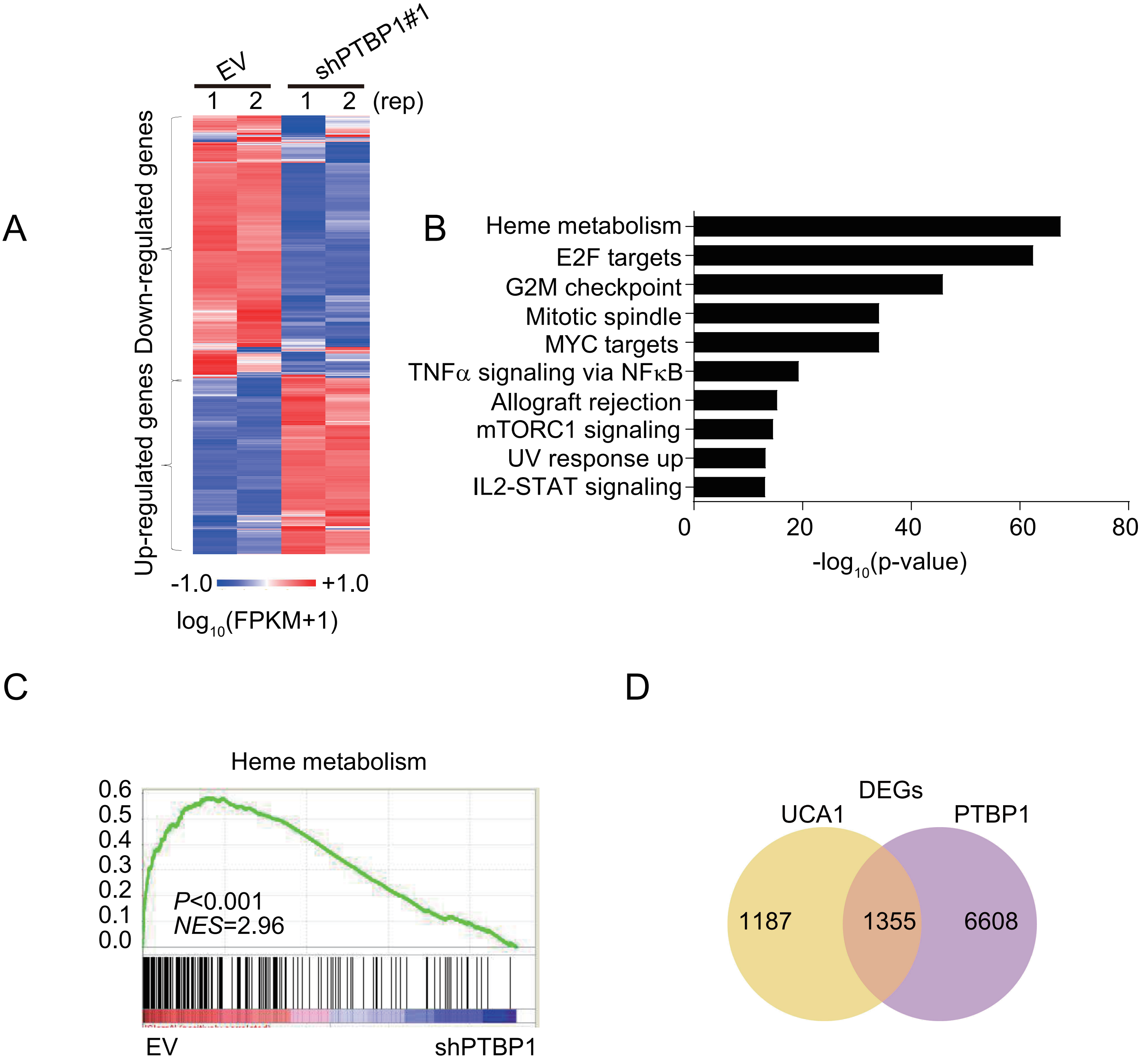

Supplementary Figure 4. **PTBP1 depletion impaired heme metabolism** (A) Heatmap depicts DEGs identified by RNA-seq on day 8 differentiated erythroid cells after PTBP1 downregulation. (B) Hallmark enrichment analysis of DEGs (top 2000) after PTBP1 knockdown. (C) GSEA showed enrichment of heme metabolism related genes after PTBP1 knockdown. Normalized enrichment scores (NES) and P values are shown. (D) The Venn diagram depicts the common DEGs identified by RNA-seq after UCA1 or PTBP1 depletion.

Supplementary Figure 5

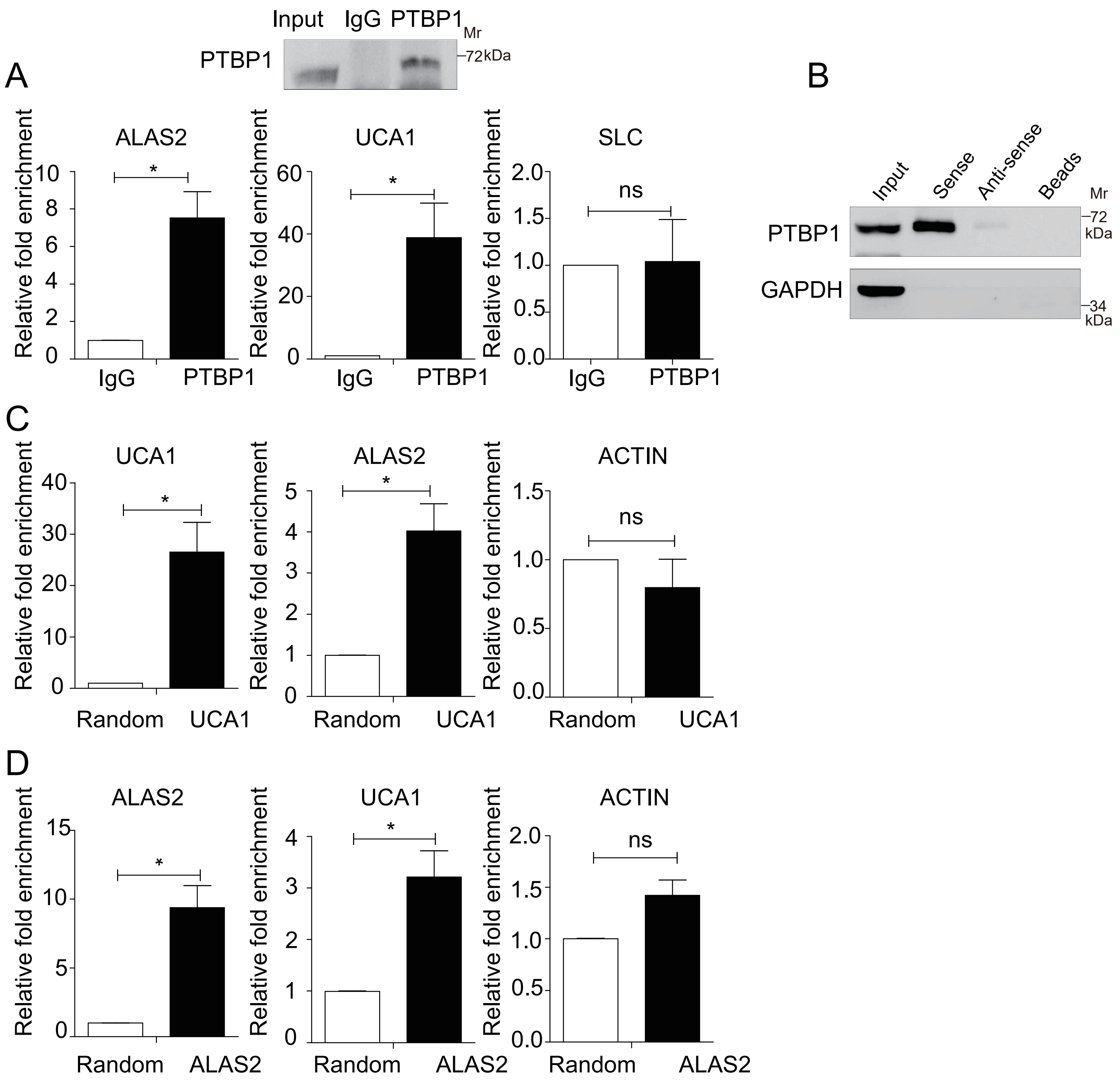

Supplementary Figure 5. UCA1 RNA, *ALAS2* mRNA and PTBP1 protein interactions in AraC-induced K562 cells (A) PTBP1 RIP assay to analyze interactions between PTBP1 protein and UCA1, *ALAS2* mRNA in AraC-induced K562 cells. WB showed PTBP1 immunoprecipitation (top). The relative fold enrichment of UCA1 or *ALAS2* mRNA compared to IgG was determined by qRT-PCR (bottom). SLC was used as a negative control. (B) *ALAS2* mRNA pull-down assay using an *in vitro* transcribed *ALAS2* mRNA also detected PTBP1 by immunoblotting in AraC-induced K562 cells. (C) *ALAS2* enrichment by the *in vivo* RNA-RNA pull-down assay using *UCA1*-specific probes and a random probe in AraC-induced K562 cells. *ACTIN* mRNA was used as a negative control. (D) *UCA1* enrichment by the *in vivo* RNA-RNA pull-down assay using *ALAS2*-specific probes and a random probe in AraC-induced K562 cells. *ACTIN* mRNA was used as a negative control.

Supplementary Figure 6

A

WT+PB UCA1$^{+/-}$+PB UCA1$^{+/-}$+ALAS2

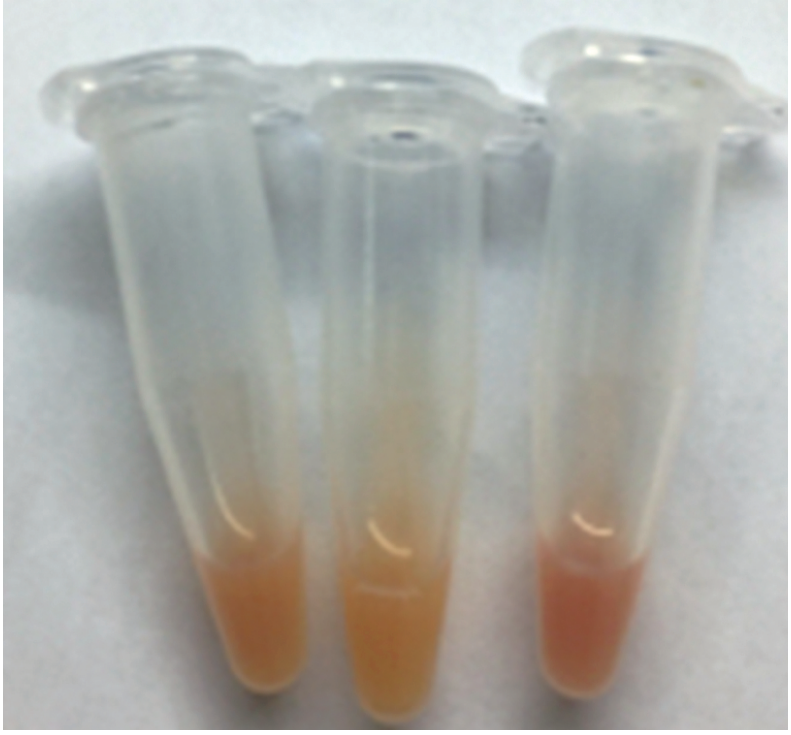

Supplementary Figure 6. The cell pellet of WT, UCA1$^{+/-}$ and UCA1$^{+/-}$ with ALAS2 overexpression K562 cells. The cells were co-transfected with pBase and piggybac transposon empty expression vector or piggybac ALAS2 expression vector. After 2 days of culture, GFP^{+} cells were isolated by FACS-based cell sorting. These sorted GFP^{+} cells were expanded and induced erythroid differentiation with 20 nM AraC for 48 h and collected.

Supplementary Figure 7

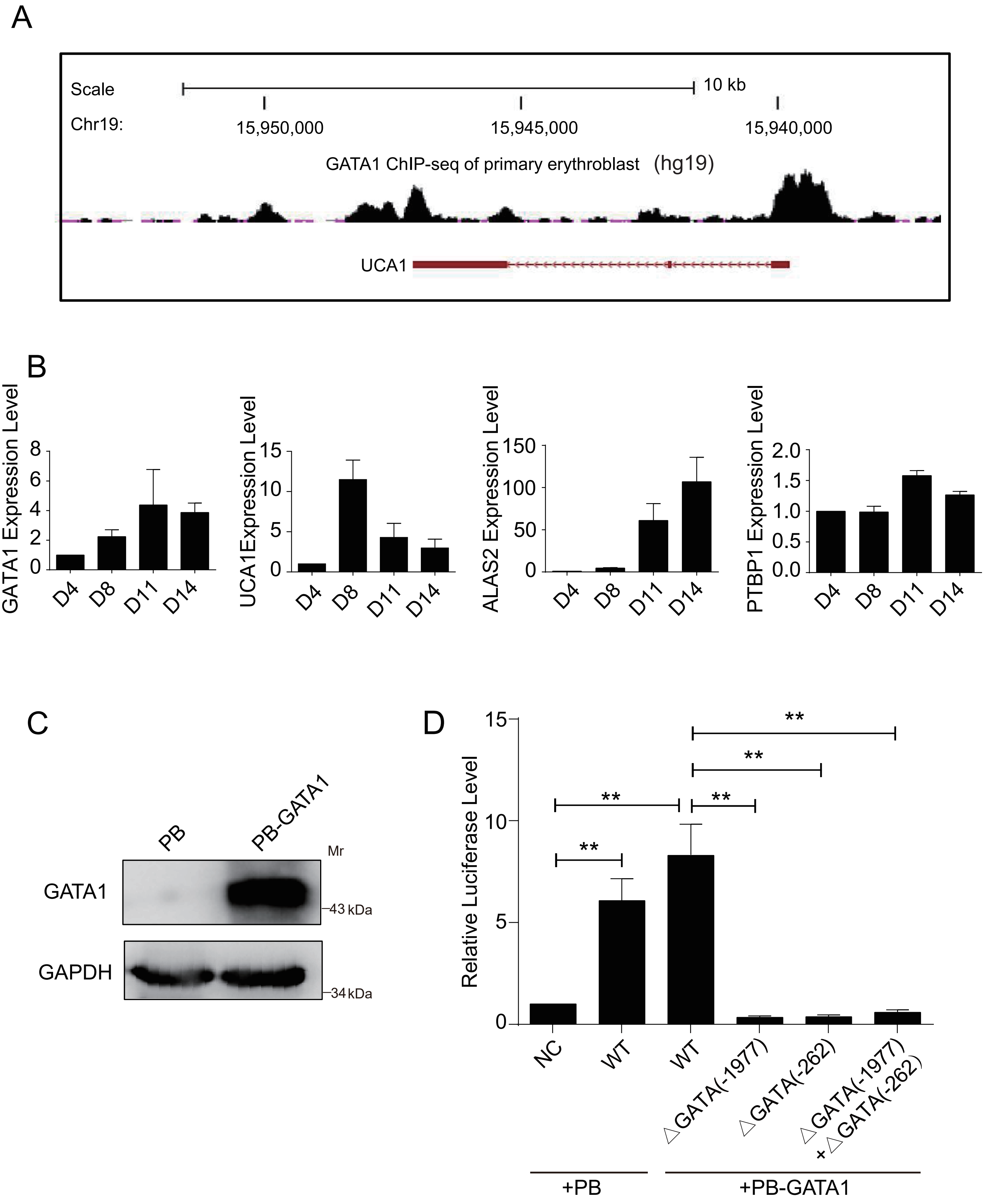

Supplementary Figure 7. GATA1 occupies the *UCA1* promoter in primary erythroid cells. (A) ChIP-Seq assay depicts GATA1 occupancy at the *UCA1* promoter region. (B) *GATA1*, UCA1, *ALAS2* and *PTBP1* expression during erythroid differentiation of cord blood $CD34^+$ cells. (C-D) Luciferase assay demonstrating GATA1 regulation of *UCA1* promoter activity in a transient transfection assay in 293T cells. GATA1 overexpression (C) increased activity of the WT-*UCA1* promoter, but not GATA motif-mutated-*UCA1* promoter (D).

Supplementary Figure 8

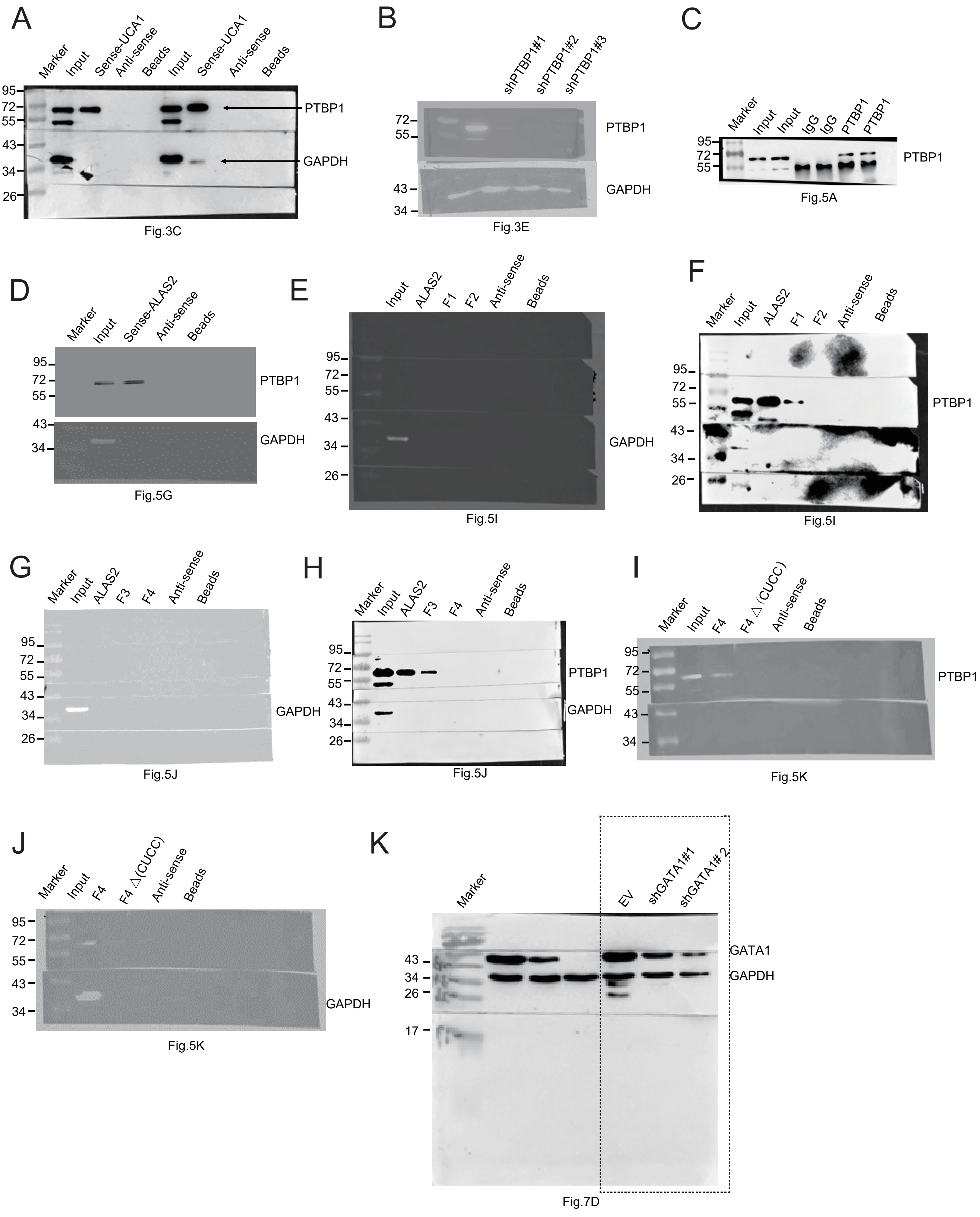

Supplementary Figure 8. Unprocessed images of western blots. (A) Figure 3C. WB using PTBP1 and GAPDH antibody, followed by UCA1 and antisense-UCA1 pull-down in HUDEP-2 cells. (B) Figure 3E. PTBP1 and GAPDH expression were examined by WB after shRNA mediated PTBP1 knockdown with day 8 differentiated primary erythroid cells. (C) Figure 5A. immunoprecipitation using an anti-PTBP1 or anti-IgG antibody and PTBP1 in immunoprecipitates was analyzed by immunoblotting. (D) Figure 5G. WB using anti-PTBP1 antibody followed by *in vitro* transcribed *ALAS2* and antisense-*ALAS2* mRNA pull-down assay in HUDEP-2 cells. GAPDH was used as a negative control. (E-J) Figure 5I-K. *In vitro* transcribed full-length or fragmental *ALAS2* mRNA pull-down assays followed by PTBP1 immunoblotting in AraC-induced K562 cells (E-H) and HUDEP-2 cells (I-K). GAPDH was used as a negative control. (K) Figure 7D. immunoblotting analysis of GATA1 and GAPDH expression at day 8 in differentiated primary erythroid cells with or without GATA1 depletion.

Supplementary Table 1.The primers used for the short hairpin RNA sequences or overexpression of ALAS2 or GATA1

Primer	Sequence 5'-3'
ShUCA1#1-F	CCGGGGTAATGTATCATCGGCTTAGCTCGAGCTAAGCCGATGATACATTACCTTTTTG
Sh UCA1#1-R	AATTcaAAAAGGTAATGTATCATCGGCTTAGCTCGAGCTAAGCCGATGATACATTACC
Sh UCA1#2-F	CCGGGGAATACTATTCGTATGATGGCTCGAGCCATCATACGAATAGTATTCCTTTTTG
Sh UCA1#2-R	AATTcaAAAAGGAATACTATTCGTATGATGGCTCGAGCCATCATACGAATAGTATTCC
Sh UCA1#3-F	CCGGGGTTCACCATTCCAGAATAAACTCGAGTTTATTCTGGAATGGTGAACCTTTTTG
Sh UCA1#3-R	AATTCAAAAAGGTTCACCATTCCAGAATAAACTCGAGTTTATTCTGGAATGGTGAACC
ShPTBP1#1-F	CCGGGCGTGAAGATCCTGTTCAATACTCGAGTATTGAACAGGATCTTCACGCTTTTTG
ShPTBP1#1-R	AATTCAAAAAGCGTGAAGATCCTGTTCAATACTCGAGTATTGAACAGGATCTTCACGC
ShPTBP1#2-F	CCGGGCCTCAACGTCAAGTACAACACTCGAGTGTTGTACTTGACGTTGAGGCTTTTTG
ShPTBP1#2-R	AATTCAAAAAGCCTCAACGTCAAGTACAACACTCGAGTGTTGTACTTGACGTTGAGGC
ShPTBP1#3-F	CCGGGCACAGTGTTGAAGATCATCACTCGAGTGATGATCTTCAACACTGTGCTTTTTG
ShPTBP1#3-R	AATTCAAAAAGCACAGTGTTGAAGATCATCACTCGAGTGATGATCTTCAACACTGTGC
ShGATA1#1-F	CCGGGAAGCGCCTGATTGTCAGTAAACGGCTCGAGCCGTTTACTGACAATCAGGCGCTTCTTTTTG
ShGATA1#1-R	AATTCAAAAAGAAGCGCCTGATTGTCAGTAAACGGCTCGAGCCGTTTACTGACAATCAGGCGCTTC
ShGATA1#2-F	CCGGGCGCCTGATTGTCAGTAAACGCTCGAGCGTTTACTGACAATCAGGCGCTTTTTG
ShGATA1#2-R	AATTCAAAAAGCGCCTGATTGTCAGTAAACGCTCGAGCGTTTACTGACAATCAGGCGC
hALAS2-F(piggybac)	GCGGATCCAAATAGACGGATAGAGCACCATGGTGACTGCAGCCATGCTGCTA
hALAS2-R(piggybac)	CGACCGGTCTTGTCATCGTCGTCCTTGTAATCGGCATAGGTGGTGACATACTG
hGATA1-F(piggybac)	ATCGACGCGTCACCATGGATTACAAGGACGACGATGACAAGGAGTTCCCTGGCCTGGGGTCCCT
h GATA1-R(piggybac)	ATCGGCTAGCTCATGAGCTGAGCGGAGCCACCACAGTA

Supplementary Table 2. The primers used for real-time PCR

Primer	Sequence 5'-3'
hACTIN-F	CTCTTCCAGCCTTCCTTCCT
hACTIN-R	AGCACTGTGTGTTGGCGTACAG
UCA1-F	AACTCTTACGGTGGAGGATTC
UCA1-R	GGTCCATTGAGGCTGTAGAGT
SLC25A21-AS1-F	GGGCAGAACTTCAGTAAGGAG
SLC25A21-AS1-R	AGCCTGAAGTCAGCTTAGTGC
ALAS2-F	TGTCCGTCTGGTGTAGTAATGA
ALAS2-R	GCTCAAGCTCCACATGAAACT
PTBP1-F	ACCAGGCCTTCATCGAGAT
PTBP1-R	GTTGGGAGAGCTGTCGGTCTT
ALAD-F	GCCTGTGTCCCTACACCTC
ALAD-R	TACCACCTGACATCCTGCCTT
PBGD-F	CTGCAAGCGGGAAAACCCT
PBGD-R	CTCCAGATGCGGGAACTTTCT
FECH-F	GGATTTCGGTACGTCCATCCT
FECH-R	TGTGGATACTGTGTGAAAGCAAT
UROS-F	GCCAAGTCAGTGTATGTGGTT
UROS-R	GCAATCCCTTTGTCCTTGAGC
HMBS-F	CTGCAAGCGGGAAAACCCT
HMBS-R	CTCCAGATGCGGGAACTTTCT
UROD-F<br>UROD-R	ATGGAAGCGAATGGGTTGGG<br>GGGAGTGTAGTCTGTTTCCTCT
CPOX-F	TGATGATCTTGACTCTCCGTCC
CPOX-R	AGTGCTTTTTCACAAGGGGAA
PPOX-F	TCTGCCGTGGAGTGTTTGC
PPOX-R	ATGGAACGATGGGTTTGCTCA
GATA-ChIP-P1-F	CCAGGCAGTTCTGAAACCCA
GATA-ChIP-P1-R	CGTTACAGGGTGATGTGACCT
GATA-ChIP-P2-F	TCACTGTGTGAGGGTTTCGG
GATA-ChIP-P2-R	ACTTGTGCTTCTCTTGTTGAGC
GATA-ChIP-P3-F	GGCTTGGGGTGAGAAAAGCTA
GATA-ChIP-P3-R	CAGCCTGAGATGTGCCTGTG

Supplementary Table 3. Primer sequences used for in vitro transcription

Primer	Sequence 5’-3’
UCA1 full length-F	ACGCACGCTGTAATACGACTCACTATAGGCTGACATTCTTCTGGACAATGAG
UCA1 full length-R	CTGACTCTTTTAGGAAGATTTC
UCA1 antisense-R	TGACATTCTTCTGGACAATGAGT
ALAS2 antisense-R	ATGGTGACTGCAGCCATGCTGCTAC
ALAS2 F1-F	ACGCACGCTGTAATACGACTCACTATAGGCATGGTGACTGCAGCCATGCTGCTAC
ALAS2 F1-R	AGGAAGGCCCCTGTCCATGAGTAGC
ALAS2 F2-R	TGCCTGCGTCTGAGTAAATCTCGCA
ALAS2 F3-F	ACGCACGCTGTAATACGACTCACTATAGGCAGATTTACTCAGACGCAGGCAACCA
ALAS2 F3-	CAGCAGCAGCTTCTCCACAAAATCT
ALAS2 F4-F	ACGCACGCTGTAATACGACTCACTATAGGCCGTGATGGAATTATGCATAAGATT
ALAS2 F4-R	TCAGGCATAGGTGGTGACATACTGG

Supplementary Table 4. The primers used for biotinylated DNA probe

Primer	Sequence 5'-3'
UCA1 probe 1#	AGCGAAGGGAGATAGGAGAGGGGC
UCA1 probe 2#	GCAAGTGGGCTGAGTCCGAAAAGA
UCA1-random probe	AGGTGAACTTCAAGATCCGCCACAA
ALAS2 probe 1#	AACAAAGCAGGAGGAGAAGAGCAGG
ALAS2 probe 2#	ATTCCATCACGCTCCCCAATCCCAG
ALAS2-random probe	GTGATGTCTAGCGCTTGGGCTTTG
